# Chitosan Enhances Low-Dosage Difenoconazole to Efficiently Control Leaf Spot Disease in *Pseudostellaria heterophylla* (Miq.) Pax

**DOI:** 10.3390/molecules28166170

**Published:** 2023-08-21

**Authors:** Cheng Zhang, Yi Dai, Jiaqi Liu, Yue Su, Qinghai Zhang

**Affiliations:** 1 Guizhou Provincial Engineering Research Center of Ecological Food Innovation, School of Public Health, Guizhou Medical University, Guiyang 550025, China; chengz76@gmc.edu.cn (C.Z.); gmcjiaqiliu@126.com (J.L.); 2 Engineering Technology Research Center for Protection and Detection of Germplasm Resources of Karst-Adaptable Crops, Department of Food and Medicine, Guizhou Vocational College of Agriculture, Qingzhen 551400, China; daiyi96@126.com

**Keywords:** leaf spot disease, chitosan, difenoconazole, *Pseudostellaria heterophylla*, declining application of pesticides

## Abstract

*Pseudostellaria heterophylla* (Miq.) Pax is a popular clinical herb and nutritious health food. However, leaf spot disease caused by fungal pathogens frequently occurs and seriously influences the growth, quality, and yield of *P. heterophylla*. In this work, the field control roles of difenoconazole, chitosan, and their combination in the leaf spot disease in *P. heterophylla* and their effects on the disease resistance, photosynthetic capacity, medicinal quality, and root yield of *P. heterophylla* are investigated. The results manifest that 37% difenoconazole water-dispersible granule (WDG) with 5000-time + chitosan 500-time dilution liquid had a superior control capacity on leaf spot disease with the control effects of 91.17%~88.19% at 15~30 days after the last spraying, which significantly (*p* < 0.05) exceeded that of 37% difenoconazole WDG 3000-time dilution liquid and was significantly (*p* < 0.01) higher than that of 37% difenoconazole WDG 5000-time dilution liquid, chitosan 500-time dilution liquid, or chitosan 1000-time dilution liquid. Simultaneously, this combination could more effectively enhance the disease resistance, photosynthetic capacity, medicinal quality, and tuberous root yield of *P. heterophylla* compared to when these elements were applied alone, as well as effectively reduce difenoconazole application. This study emphasizes that chitosan combined with a low dosage of difenoconazole can be proposed as a green, efficient, and alternative formula for controlling leaf spot disease in *P. heterophylla* and enhancing its resistance, photosynthesis, quality, and yield.

## 1. Introduction

*Pseudostellaria heterophylla* (Miq.) Pax, a popular clinical herb and nutritious health food, is widely distributed in China, Japan, the Russian Far East, South Korea, and North Korea [1]. Its dried tuberous root is rich in polysaccharides, saponins, cyclic peptides, flavonoids, amino acids, and minerals, and has high medicinal, nutritional, and commercial values [2,3,4]. As a traditional Chinese medicine and ginseng substitute, it has various pharmacological functions, such as invigorating the spleen and stomach, replenishing qi, moistening the lungs, benefiting the blood, protecting myocardial function, enhancing immunity, and inhibiting tumor cells, as well as anti-fatigue, anti-inflammation, anti-aging, anti-cough, and anti-virus properties [4,5,6,7]. Most notably, it was also a representative Chinese medicine for the prevention and treatment of coronavirus disease 2019 (COVID-19) due to its beneficial functions [8]. Currently, as a rural revitalization and enrichment industry, it is widely planted in the Guizhou, Fujian, Jiangsu, and Anhui provinces in China, especially in Guizhou, where the planting area exceeds 20,000 hm^2^ [9]. Despite the continuous expansion of commercial cultivation, leaf spot disease caused by fungal pathogens including *Alteraria tenuissima*, *Phyllosticta commonsii*, *Septoria* sp., *Arcopilus versabilis*, and *Phoma* sp. frequently occurs and seriously influences the growth, quality, and yield of *P. heterophylla*, as well as constantly causing over 50% of economic losses [10,11,12,13]. Consequently, some scholars proposed that some chemical or biological fungicides and biocontrol bacteria could be used for controlling leaf spot disease, such as flusilazole, azoxystrobin, pyraclostrobin, shenqinmycin, eugenol, and *Bacillus subtilis* [10,11,12,13,14]. Considering the serious harmfulness of leaf spot disease, it is essential to develop various candidates and practicable control technologies to improve the quality and productivity of *P. heterophylla*.

Difenoconazole, a systemic triazole fungicide, is one of the most long-term and mainstream applied fungicides in the world [15,16]. Due to its high effectiveness, high persistence, broad spectrum, and low toxic properties, it is very effective to protect fruits, vegetables, and crops from fungal diseases, such as anthracnose, scab, false smut, powdery mildew, etc. [17,18,19,20]. It disrupts the structure and function of cell membranes in pathogens by inhibiting ergosterol biosynthesis [21]. For leaf spot disease in *P. heterophylla*, He et al. [10] found that 37% difenoconazole water-dispersible granule (WDG) had a superior antifungal activity against *Alteraria tenuissima* with an EC_50_ value of 27.31 μg mL^−1^. Li et al. [13] reported that the inhibitory rate of 37% difenoconazole WDG 2000-time dilution liquid on *Arcopilus versabilis* mycelium was higher than 90%. Li et al. [22] showed that the 10% difenoconazole WDG had a 76.54% control effect on leaf spot disease caused by *Phyllostatita commonsii*. Although difenoconazole has excellent control potential in leaf spot disease in *P. heterophylla*, it is substantially and permanently applied to *P. heterophylla* fields, which might bring several hidden troubles to human health, animals, hydrobios, and the ecosystem [23,24,25,26]. Meanwhile, the resistance of pathogenic fungi to difenoconazole is also easily triggered by the increase in application frequency [27]. Notably, our previous report found that chitosan can be applied as an effective adjuvant to promote the low-dosage use of pyraclostrobin against powdery mildew in *Rose roxburghii,* to improve its resistance, photosynthesis, yield, quality, and amino acid content, as well as to reduce pyraclostrobin application [28]. In this case, screening some natural biomolecules as difenoconazole adjuvants for the highly effective control of leaf spot disease in *P. heterophylla*, a decline in difenoconazole application and its hidden troubles, and delayed pathogen resistance are worthy of further attention.

Chitosan, a natural non-toxic biomolecule from chitin deacetylation, is widely used as a growth promoter, resistance inducer, biological fungicide, or fertilizer in agricultural production [29,30,31,32,33]. Moreover, it has favorable nontoxic, biocompatible, biodegradable, and renewable properties so that it can also be widely applied in food, medicine, and cosmetic fields [34,35]. Many researchers have reported the beneficial effects of chitosan on the growth, quality, and yield of crops, fruits, vegetables, and traditional Chinese medicines. For instance, Liu et al. [36] found that the growth, photosynthesis, resistance, yield, and quality of *Platycodon grandifloras* treated with chitosan soaking were effectively improved, and they found that chitosan also promoted the drought resistance of *Sctellaria baicalensis* seedlings [37], induced *R. roxburghii* against powdery mildew, and enhanced its photosynthesis and quality [38]. Our previous report also indicated that chitosan spraying effectively enhanced the growth, photosynthesis, resistance, yield, and quality of the continuous cropping of *Pinellia ternata* [39]. Meanwhile, chitosan can be applied as an effective adjuvant to promote isopyrazam·azoxystrobin and tetramycin, or pyraclostrobin, allicin, and physcion to control kiwifruit leaf spot or *Rosa roxburghii* powdery mildew, and reduce their application dosage [28,40,41,42,43]. Overall, whether chitosan can ameliorate the control effect of difenoconazole on leaf spot disease in *P. heterophylla* and whether their co-application can more effectively enhance the growth, quality, and yield of *P. heterophylla* is also worth further study.

This study evaluated the field control effects of difenoconazole, chitosan, and their combination on leaf spot disease in *P. heterophylla*. Subsequently, the influences of difenoconazole, chitosan, and their combination on the disease resistance and photosynthetic characteristics of *P. heterophylla* leaves were investigated, as were the influences of difenoconazole, chitosan, and their combination on the medicinal quality and yield properties of *P. heterophylla* roots. This work aimed to provide an alternative natural biomolecule-assisted fungicide technology to control leaf spot disease in *P. heterophylla* and reduce chemical pesticide application.

## 2. Results

### 2.1. Field Control Effects of Difenoconazole and Chitosan on Leaf Spot Disease

The field control effects of difenoconazole and chitosan on leaf spot disease in *P. heterophylla* are shown in Table 1. D 5000 + C 500, D 3000, D 5000, C 500, and C 1000 significantly (*p* < 0.01) diminished the disease index of leaf spot disease in *P. heterophylla* 15 days and 30 days after the last spraying. D 3000 and D 5000 had good control capacities on leaf spot disease in *P. heterophylla,* with control effects of 89.30~80.13% and 75.44~69.92% at 15 days and 30 days after the last spraying, respectively. Although chitosan exhibited a relatively inferior control potential compared with difenoconazole, the control effects of C 500 and C 1000 on leaf spot disease in *P. heterophylla* reached 62.96~57.85% and 56.70~53.07% at 15 days and 30 days after the last spraying, respectively. D 5000 + C 500 displayed an optimal control capacity on leaf spot disease in *P. heterophylla*, with control effects of 91.17% and 88.19% at 15 days and 30 days after the last spraying, respectively, which significantly (*p* < 0.05) exceeded those of D 3000 and were significantly (*p* < 0.01) higher than those of D 5000, C 500, and C 1000. Simultaneously, the difenoconazole application dosage of D 5000 + C 500 effectively declined compared with that of D 3000. These results indicate that chitosan had a favorable induced control effect on leaf spot disease in *P. heterophylla*, and combined with low-dosage difenoconazole, it was more effective at controlling leaf spot disease in *P. heterophylla* than high-dosage difenoconazole, as well as effectively reducing the application dosage of difenoconazole.

### 2.2. Influences of Difenoconazole and Chitosan on Disease Resistance in P. heterophylla Leaves

**Figure 1:**
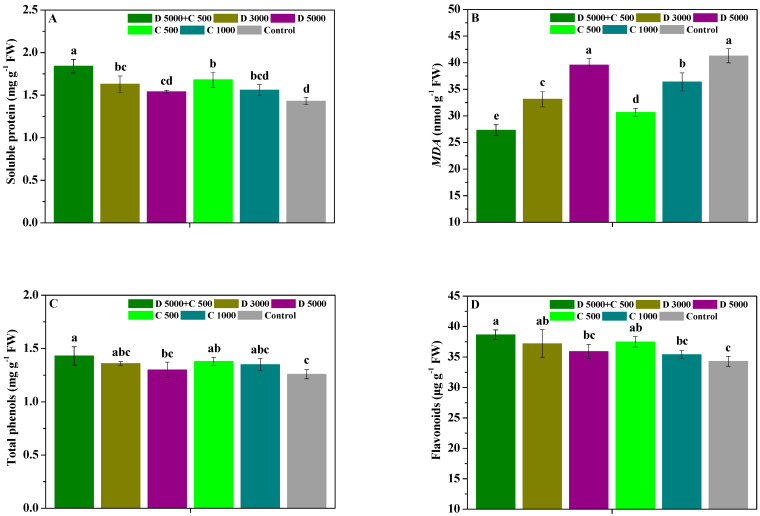
The influences of difenoconazole and chitosan on the soluble protein (**A**), MDA (**B**), total phenols (**C**), and flavonoid (**D**) contents in *P. heterophylla* leaves. Error bar represents SD; small letters indicate significant differences at 5% (*p* < 0.05) level.

The influences of difenoconazole and chitosan on the soluble protein MDA, total phenols, and flavonoids in *P. heterophylla* leaves are displayed in Figure 1. Compared with the control, D 5000 + C 500, D 3000, and C 500 significantly (*p* < 0.05) increased the soluble protein, total phenols, and flavonoid contents in *P. heterophylla* leaves; D 5000 and C 1000 slightly increased the soluble protein, total phenols, and flavonoid contents in *P. heterophylla* leaves; and D 5000 + C 500, D 3000, D 5000, C 500, and C 1000 significantly (*p* < 0.05) decreased their MDA contents. Moreover, the soluble protein content in *P. heterophylla* leaves treated with D 5000 + C 500 was significantly (*p* < 0.05) higher than that in leaves treated with D 3000, D 5000, C 500, or C 1000; their MDA content was significantly (*p* < 0.05) lower than that in leaves treated with D 3000, D 5000, C 500, and C 1000; their total phenol content was significantly (*p* < 0.05) higher than that in leaves treated with D 5000; and their flavonoid content was significantly (*p* < 0.05) higher than that in leaves treated with D 5000 and C 1000. However, there were no significant (*p* < 0.05) differences in the soluble protein, total phenols, and flavonoid contents in *P. heterophylla* leaves between D 3000 and D 5000, or C 500 and C 1000, but those for leaves treated with D 3000 and C 500 were slightly higher than those for leaves treated with D 5000 and C 1000, respectively. These results show that chitosan combined with difenoconazole can effectively promote the soluble protein, total phenols, and flavonoid contents in *P. heterophylla* leaves and decline their MDA content, thereby enhancing their disease resistance to leaf spot disease.

**Figure 2:**
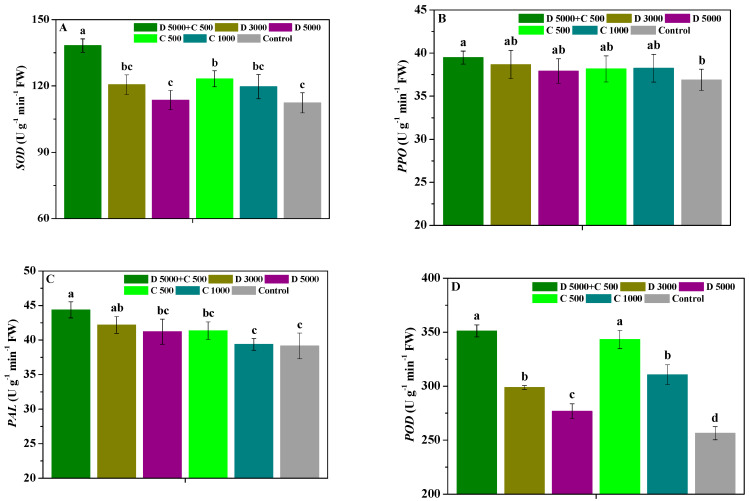
The influences of difenoconazole and chitosan on the SOD (**A**), PPO (**B**), PAL (**C**), and POD (**D**) activities in *P. heterophylla* leaves. Error bar represents SD; small letters indicate significant differences at 5% (*p* < 0.05) level.

The influences of difenoconazole and chitosan on the SOD, PPO, PAL, and POD activities in *P. heterophylla* leaves are shown in Figure 2. Compared with the control, D 5000 + C 500 significantly (*p* < 0.05) enhanced the SOD, PPO, PAL, and POD activities in *P. heterophylla* leaves; C 500 significantly (*p* < 0.05) promoted their SOD activity; D 5000 significantly (*p* < 0.05) enhanced their PAL activity; and D 3000, D 5000, C 500, and C 1000 significantly (*p* < 0.05) improved their POD activity. Furthermore, the SOD activity in *P. heterophylla* leaves treated with D 5000 + C 500 was significantly (*p* < 0.05) higher than that in leaves treated with D 3000, D 5000, C 500, and C 1000; their PPO activity was slightly higher than that in leaves treated with D 3000, D 5000, C 500, and C 1000; their PAL activity was significantly (*p* < 0.05) higher than that in leaves treated with D 5000, C 500, and C 1000; and their POD activity was significantly (*p* < 0.05) higher than that in leaves treated with D 3000, D 5000, and C 1000. Moreover, the POD activity in *P. heterophylla* leaves treated with D 3000 and C 500 was significantly (*p* < 0.05) higher than that in leaves treated with D 5000 and C 1000, respectively. Meanwhile, the SOD, PPO, and PAL activities in *P. heterophylla* leaves were not significantly (*p* < 0.05) different between those treated with D 3000 and D 5000, or C 500 and C 1000, but those treated with D 3000 and C 500 showed slightly higher activities than those treated with D 5000 and C 1000, respectively. These results show that difenoconazole in combination with chitosan can effectively improve the enhancing effects of their treatment alone on the SOD, PPO, PAL, and POD activities in *P. heterophylla* leaves, reliably promoting the disease resistance of *P. heterophylla*.

### 2.3. Influences of Difenoconazole and Chitosan on Photosynthetic Capacity in P. heterophylla Leaves

**Figure 3:**
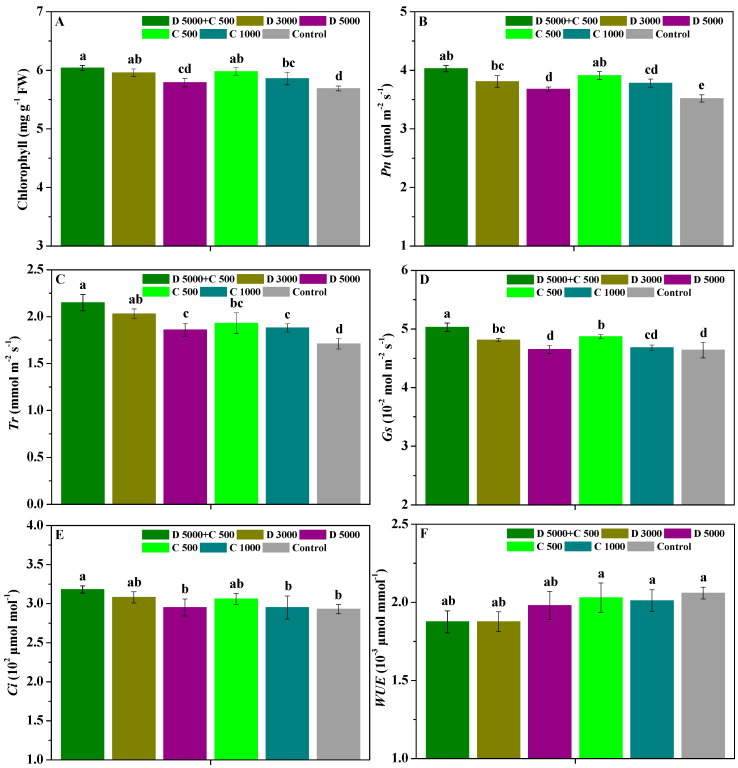
The influences of difenoconazole and chitosan on the chlorophyll (**A**), Pn (**B**), Tr (**C**), Gs (**D**), Ci (**E**), and WUE (**F**) in *P. heterophylla* leaves. Error bar represents SD; small letters indicate significant differences at 5% (*p* < 0.05) level.

The influences of difenoconazole and chitosan on the chlorophyll, Pn, Tr, Gs, Ci, and WUE contents in *P. heterophylla* leaves are shown in Figure 3. Compared with the control, D 5000 + C 500, D 3000, C 500, and C 1000 significantly (*p* < 0.05) enhanced the chlorophyll content in *P. heterophylla* leaves; D 5000 + C 500, D 3000, D 5000, C 500, and C 1000 significantly (*p* < 0.05) enhanced their Pn and Tr contents; D 5000 + C 500, D 3000, D 5000, and C 500 significantly (*p* < 0.05) improved their Gs content; and D 5000 + C 500 significantly (*p* < 0.05) increased their Ci content. Moreover, the chlorophyll, Pn, Tr, Gs, and Ci contents in *P. heterophylla* leaves treated with D 5000 + C 500 were 6.04 mg g^−1^, 4.03 μmol m^−2^ s^−1^, 2.15 mmol m^−2^ s^−1^, 5.03 × 10^−2^ mol m^−2^ s^−1^, and 3.18 × 10^2^ μmol mol^−1^, which were increased by 1.01, 1.04, 1.01, and 1.03 fold; 1.06, 1.10, 1.03, and 1.07 fold; 1.06, 1.16, 1.11, and 1.14 fold; 1.05, 1.08, 1.03, and 1.07 fold; and 1.03, 1.08, 1.04, and 1.08 fold compared with D 3000, D 5000, C 500, and C 1000, respectively. Additionally, the chlorophyll, Pn, Tr, and Gs contents in *P. heterophylla* leaves treated with D 3000 were significantly (*p* < 0.05) higher than in those treated with D 5000, and the Pn and Gs contents in *P. heterophylla* leaves treated with C 500 were significantly (*p* < 0.05) higher than in those treated with C 1000. Nevertheless, the WUE content in *P. heterophylla* leaves was not significantly (*p* < 0.05) different between all treatments. The results demonstrate that chitosan used in combination with low-dosage difenoconazole can effectively promote the chlorophyll, Pn, Tr, Gs, and Ci contents in *P. heterophylla* leaves compared with treatments using difenoconazole or chitosan alone, favoring growth.

### 2.4. Influences of Difenoconazole and Chitosan on Quality and Yield in P. heterophylla

The influences of difenoconazole and chitosan on the ash, extractum, polysaccharide, and total saponin contents in *P. heterophylla* roots are shown in Table 2. Compared with the control, D 5000 + C 500, D 3000, and C 500 significantly (*p* < 0.05) improved the ash, extractum, and polysaccharide contents in *P. heterophylla* roots, and D 5000 + C 500, D 3000, D 5000, C 500, and C 1000 significantly (*p* < 0.05) enhanced their total saponin contents. Moreover, the ash and extractum contents in *P. heterophylla* roots treated with D 5000 + C 500 were significantly (*p* < 0.05) higher than in those treated with D 5000 and C 1000, and their polysaccharide and total saponin contents were significantly (*p* < 0.05) higher than in those treated with D 3000, D 5000, C 500, and C 1000. Simultaneously, the ash, extractum, and total saponin contents in *P. heterophylla* roots treated with D 3000 and C 500 were significantly (*p* < 0.05) higher than in those treated with D 5000 and C 1000, respectively, and their polysaccharide contents were not significantly (*p* < 0.05) different from those in roots treated with D 3000, D 5000, C 500, and C 1000. These findings show that the co-application of chitosan and difenoconazole can more effectively improve the medicinal qualities of *P. heterophylla* roots.

**Figure 4:**
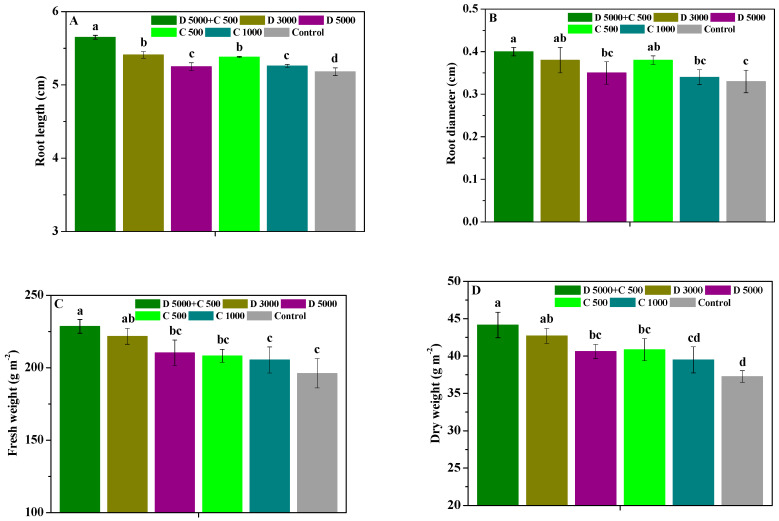
The influences of difenoconazole and chitosan on root length (**A**), root diameter (**B**), fresh weight (**C**), and dry weight (**D**) in *P. heterophylla* roots. Error bar represents SD; small letters indicate significant differences at 5% (*p* < 0.05) level.

The influences of difenoconazole and chitosan on the root length, root diameter, fresh weight, and dry weight of *P. heterophylla* roots are shown in Figure 4. Compared with the control, D 5000 + C 500, D 3000, D 5000, C 500, and C 1000 effectively increased the root length, root diameter, fresh weight, and dry weight of *P. heterophylla* roots. D 5000 + C 500 increased the yield capability of *P. heterophylla* with a root length, root diameter, fresh weight, and dry weight of 5.65 cm, 0.40 cm, 228.54 g m^−2^, and 44.16 g m^−2^, which were increased by 1.04, 1.08, 1.05, 1.07, and 1.09 fold; 1.05, 1.14, 1.05, 1.18, and 1.21 fold; 1.03, 1.09, 1.10, 1.01, and 1.17 fold; 1.03, 1.09, 1.08, 1.12, and 1.19 fold; and 1.03, 1.08, 1.04, and 1.08 fold compared with D 3000, D 5000, C 500, C 1000, and the control, respectively. The results show that using chitosan combined with low-dosage difenoconazole can more effectively enhance *P. heterophylla*’s root growth and yield increase.

## 3. Materials and Methods

### 3.1. Fungicide, Chitosan, and Chemicals

Difenoconazole WDG of 37% was obtained from Jiangxi Heyi Chemical Co., Ltd. (Jiuan, China). Chitosan with more than 90% deacetylation was purchased from Mingrui Bioengineering Co., Ltd. (Zhenzhou, China). The other chemicals were of chromatographic or analytical grade.

### 3.2. Field Herbal Garden

The field control experiment on leaf spot disease in *P. heterophylla* was carried out in a *P. heterophylla* herbal garden in Wujiatang village, Niudachang Town, Shibing country, Guizhou Province, China (27°14′ N, 107°97′ E). *P. heterophylla* of the ‘Shitai 1’ cultivar was cultivated via ridging, where each plot area was 3.0 m^2^ (with a ridge width of 6.0 m, length of 0.5 m, and ridge of 0.2 m) and the application dosage of seed roots was 300 kg per 667 m^2^. The previous crop in the herbal garden was also *P. heterophylla*. The annual sunshine, annual rainfall, average temperature, average altitude, and frostless season in the *P. heterophylla* herbal garden were 1197 h, 1060 mm, 15.0 °C, 943 m, and 274.5 d, respectively. Additionally, Table 3 displays the soil fertility of the *P. heterophylla* herbal garden.

### 3.3. Control Experiment on Leaf Spot Disease

A completely randomized method and the foliar spray method were applied to delineate the experimental plots and spray the fungicide or chitosan dilution liquid, respectively. Six treatments were projected to control leaf spot disease in *P. heterophylla*: (1) a 37% difenoconazole WDG 5000-times + chitosan 500-times dilution liquid (D 5000 + C 500), (2) a 37% difenoconazole WDG 3000-times dilution liquid (D 3000), (3) a 37% difenoconazole WDG 5000-times dilution liquid (D 5000), (4) a chitosan 500-times dilution liquid (C 500), (5) a chitosan 1000-times dilution liquid (C 1000), and (6) clear water (control). Each treatment had 3 replicates with a total of 18 plots. Fungicide or chitosan dilution liquid was sprayed on the above-ground parts of *P. heterophylla* plants using an electrostatic atomizer (Qiming Machinery Co. Ltd., Taizhou, China) on 26 March, 2 April, and 9 April, respectively. The application amount of dilution liquid was 60 L per 667 m^2^.

### 3.4. Analytical Method

#### 3.4.1. Disease Index and Control Effect

The disease index of leaf spot disease in P. heterophylla was investigated on 24 April (15 days after the last spraying) and 9 May (30 days after the last spraying) as described by Wang et al. [44], with slight modifications. Six *P. heterophylla* plants were randomly selected from each area in the east, west, south, north, and central areas in each plot, and a total of thirty plants were used to investigate the total leaf number and diseased leaf number. The incidence grades were classified as follows: grade 0—no disease spots; grade 1—the disease spots are small and few, and their area accounts for less than 5% of the whole leaf area; grade 3—the disease spots are small and numerous, or large and few, and their area accounts for 6~10% of the whole leaf area; grade 5—the disease spots are large and numerous, and their area accounts for 11~20% of the whole leaf area; grade 7—the disease spots are large and numerous, or multiple disease spots are connected to form large disease spots, and their area accounts for 21~50% of the whole leaf area; and grade 9—the disease spot area accounts for more than 51% of the whole leaf area. Furthermore, Equations (1) and (2) were used to calculate the disease index and control effect of leaf spot disease in *P. heterophylla*, respectively.

Disease index = 100 × ∑ (Grade value × Leaf number within each grade)/(Total leaf number × the highest grade)(1)

Control effect (%) = 100 × (Disease index of control − Disease index of treatment)/Disease index of control(2)

#### 3.4.2. Disease Resistance Parameters

Fresh leaves of the same orientation were collected and taken to the laboratory to determine the disease resistance parameters of *P. heterophylla* on 9 May, as described by Wang et al. [40] and Zhang et al. [41,45]. The soluble protein and malonaldehyde (MDA) contents were measured using the Coomassie Brilliant Blue method and the thiobarbituric acid (TBA) method, respectively. An amount of 1.0 g of *P. heterophylla* leaves was ground and homogenized with 10 mL of 80% methanol, extracted in a refrigerator at 4 °C for 24 h, and then centrifuged at 5600× *g* for 10 min. The supernatant was kept at −20 °C to determine the total phenols and flavonoid contents. An amount of 5.0 mL of water, 1 mL of Folin’s reagent, and 1.5 mL of a 20% sodium carbonate solution were successively added to 1.0 mL of the supernatant, shaken well, and then sheltered from light at 35 °C for 0.5 h. The light absorption value was determined at 765 nm, corresponding to the gallic acid standard curve to determine the total phenol content in *P. heterophylla* leaves. An amount of 7 mL of methanol was added to 1 mL of the supernatant in a centrifuge tube, and 6 mL of methanol and 1 mL of a 2% zirconium dioxide methanol solution were added to 1 mL of the supernatant in another centrifuge tube, after which they were mixed and bathed in water at 30 °C for 60 min. The light absorption values of the two tubes were measured at 420 nm, taking their difference as corresponding to the rutin standard curve to measure the flavonoid content in *P. heterophylla* leaves. Simultaneously, the superoxide dismutase (SOD), polyphenoloxidase (PPO), phenylalaninammonialyase (PAL), and peroxidase (POD) activities in *P. heterophylla* leaves were measured using the nitrogen blue tetrazole, catechol, trans-cinnamic acid, and guaiacol methods, respectively.

#### 3.4.3. Photosynthesis Parameters

Healthy leaves of the same orientation were selected on 9 May to measure their photosynthesis parameters according to Chen et al. [39] and Zhang et al. [41]. The photosynthetic rate (Pn), transpiration rate (Tr), stomatal conductance (Gs), intercellular carbon dioxide concentration (Ci), and water use efficiency (WUE) were monitored using a portable LI-6400XT photosynthesis measurement system (LI-COR Inc., Lincoln, NE, USA) with photosynthetically active radiation of 1000 μmol m^−2^ s^−1^ at 8:00~10:00 a.m. Meanwhile, the chlorophyll a and chlorophyll b contents of *P. heterophylla* leaves were determined with acetone–ethanol (*v*/*v*, 2:1) extraction using a UV-5800PC spectrophotometer at 663 nm and 645 nm, respectively. The chlorophyll content in *P. heterophylla* leaves was the sum of the chlorophyll a and chlorophyll b contents.

#### 3.4.4. Yield and Quality Parameters

The underground roots of *P. heterophylla* in each plot were collected and washed on 30 June. The average length and diameter of 100 roots in each plot were measured using a ruler and vernier scale, and the fresh weight and dry weight of the *P. heterophylla* roots in each plot were also determined using the gravimetric method. Moreover, the ash, extractum, polysaccharide, and total saponin contents in the *P. heterophylla* root were checked according to the general principles of four parts of the Chinese Pharmacopoeia 2020 [46].

### 3.5. Statistical Analyses

All data are displayed as mean values ± standard deviation (SD). The significant differences in the data were analyzed using Duncan’s test with one-way analysis of variance (ANOVA) with SPSS 18.0 software (SPSS Inc., Chicago, IL, USA). Origin 10.0 software (OriginLab, Northampton, MA, USA) was applied to draw the figures.

## 4. Discussion

Difenoconazole is a systemic, broad-spectrum, and mainstream triazole fungicide that can inhibit the ergosterol biosynthesis in the fungal pathogenic cell membranes of various plant diseases such as anthracnose, scab, false smut, powdery mildew, etc. [17,18,19,20]. Three previous reports indicated that difenoconazole had a positive control potentiality on leaf spot disease in *P. heterophylla* caused by *Alteraria tenuissima*, *Arcopilus versabilis*, and *Phyllostatita commonsii* [10,13,22]. Meanwhile, chitosan can be used as a resistance inducer or biological fungicide for various plant diseases due to its favorable antifungal activity [47,48,49,50]. For instance, chitosan had an inducing control effect of 69.30%~72.87% on powdery mildew in *R. roxburghii* [38] and induced kiwifruit resistance against leaf spot disease caused by *Alternaria tenuissima* and *Lasiodiplodia theobromae* with a control effect of 56.61% [41]. In this study, the 37% difenoconazole WDG 5000-times + chitosan 500-times dilution liquid exhibited an optimal control capacity on leaf spot disease in *P. heterophylla*, with control effects of 91.17% and 88.19% at 15 days and 30 days after the last spraying, respectively, which significantly (*p* < 0.05) exceeded those of the 37% difenoconazole WDG 3000-times dilution liquid and was significantly (*p* < 0.01) higher than those of the 37% difenoconazole WDG 5000-timex dilution liquid, chitosan 500-times dilution liquid, and chitosan 1000-times dilution liquid. These results indicate that the control effect of difenoconazole on leaf spot disease in *P. heterophylla* was significantly enhanced with chitosan, and their co-application effectively reduced the application dosage of difenoconazole. The mechanism of this synergistic effect may derive from difenoconazole’s and chitosan’s abilities to prevent and kill fungal pathogens and chitosan’s ability to induce plants’ disease resistance. 

Proteins are the life activity basis of plants, and the disease-course-related proteins plants contain are considered an important indicator of induced resistance. MDA also reflects the lipid peroxidation degree of cell membranes [38,41,51]. Phenols and flavonoids are two important phytoalexins that participate in lignin biosynthesis and can enhance the ligninization of host cells to produce disease resistance [51]. SOD can reduce the damage of reactive oxygen species to plant cell membranes, PPO is closely related to lignin biosynthesis, and PAL is a key enzyme in the phenylpropanoid metabolism pathway and closely related to the synthesis of defense substances such as lignin and plant phytoalexins. Meanwhile, POD can catalyze the oxidation of many substrates in the presence of H_2_O_2_ [38,41,51]. As a resistance inducer of plants, chitosan can promote increases in disease resistance substances and stimulate the activity of defense enzymes, as confirmed in many reports [30,31,32,47,48,49,50]. In addition, difenoconazole can raise antioxidant contents, reduce MDA contents, and promote proline synthesis [52]. In this study, chitosan combined with difenoconazole could more effectively enhance the soluble protein, total phenols, and flavonoid contents as well as SOD, PPO, PAL, and POD activities in *P. heterophylla* leaves and reduce their MDA content compared with the application of difenoconazole or chitosan alone, thereby more reliably improving the disease resistance of *P. heterophylla* against leaf spot disease. These results emphasize that the induced disease resistance performance of chitosan had a positive role in the efficient control of leaf spot disease via the combination of difenoconazole and chitosan.

Notably, triazole fungicides have a good growth-regulating effect on plants, which can effectively enhance plants’ photosynthesis and increase carbohydrate accumulation and crop yields [53,54]. For example, Zheng et al. [10] found that applying difenoconazole to tomato plants increased their soluble sugar content, reduced their organic acid content, and increased the accumulation of nutrient-related metabolites during late fruit ripening. As a growth promoter and biological fertilizer of plants, many researchers have confirmed that chitosan can promote the growth, photosynthesis, quality, and yield of various plants, such as *R. roxburghii*, *P. Grandiflorus*, *S. baicalensis*, *P. ternata*, *Actinidia chinensis*, etc. [28,36,37,38,39,40]. Chakraborty et al. [29] indicated that chitosan can enhance plants’ photosynthesis by raising the chlorophyll content and further promote their growth and development. The results in this study demonstrate that chitosan used with low-dosage difenoconazole more effectively promoted the chlorophyll, Pn, Tr, Gs, and Ci contents in *P. heterophylla* leaves compared with treatment using high-dosage difenoconazole or chitosan alone, which was related to the protection of *P. heterophylla* from leaf spot diseases via the combination of difenoconazole and chitosan and their growth promotion effects. The unique medicinal and nutritional functions of *P. heterophylla* are determined by the various active ingredients in their tuberous roots, including polysaccharides, saponins, and minerals [55]. In this study, the findings show that the co-application of chitosan and difenoconazole more effectively improved the ash, extractum, polysaccharide, and saponin contents in *P. heterophylla* roots and their root length, root diameter, fresh weight, and dry weight. These results emphasize that chitosan is an effective natural adjuvant of low-dosage difenoconazole in enhancing the medicinal quality, root growth, and yield increase of *P. heterophylla*.

In recent years, the reduced use of chemical pesticides and supplemental or alternative methods for plant disease management have attracted increasing attention [40,41,56]. Some reports have indicated that chitosan could be applied as an efficient adjuvant to enhance isopyrazam·azoxystrobin, tetramycin, pyraclostrobin, allicin, and physcion to control leaf spot and powdery mildew in various plants [28,40,41,42,43]. The present study found that compared with treatment using high-dosage difenoconazole or chitosan alone, chitosan used with low-dosage difenoconazole more effectively controlled leaf spot disease in *P. heterophylla* and enhanced its disease resistance, photosynthesis, quality, and yield, as well as effectively reducing difenoconazole application. The possible mechanism of these positive synergistic effects is as follows: Difenoconazole can prevent and kill fungal pathogens and can be applied as a growth enhancer to promote the photosynthesis, carbohydrate accumulation, and root yield of *P. heterophylla*. Chitosan can induce the disease resistance of *P. heterophylla* and enhance its growth, photosynthesis, quality, and yield. Furthermore, the difenoconazole dosage in combination with chitosan is very low (5000-times dilution liquid), chitosan is natural and non-toxic, and the safe interval time of 80 days is very long. Hence, the potential food risk caused by their combination is almost nonexistent. This work proposes the 37% difenoconazole WDG 5000-times + chitosan 500-times dilution liquid as a feasible candidate formula for controlling leaf spot disease in *P. heterophylla* and reducing chemical pesticide application.

## 5. Conclusions

In conclusion, the control effect of low-dosage difenoconazole on leaf spot disease in *P. heterophylla* was effectively improved using chitosan. Moreover, chitosan combined with low-dosage difenoconazole could more effectively enhance the disease resistant substance contents and resistant enzyme activities of *P. heterophylla* leaves compared to their alone application. Meanwhile, the co-application of chitosan and difenoconazole effectively improved the enhancing effects of their treatment alone on the photosynthetic capacity, medicinal quality, and tuberous root yield of *P. heterophylla*. This work highlights that chitosan combined with low-dosage difenoconazole can be used as an efficient and green candidate formula for controlling leaf spot disease in *P. heterophylla* and reducing chemical pesticide application.

## Figures and Tables

**Table 1:** Field control effects of difenoconazole and chitosan on leaf spot disease in *P. heterophylla*.

Treatments	15 Days after the Last Spraying		30 Days after the Last Spraying	
	Disease Index	Control Effect (%)	Disease Index	Control Effect (%)
D 5000 + C 500	0.47 ± 0.07 ^dD^	91.17 ± 1.75 ^aA^	0.78 ± 0.16 ^eE^	88.19 ± 1.87 ^aA^
D 3000	0.84 ± 0.10 ^dCD^	84.28 ± 2.26 ^bA^	1.30 ± 0.26 ^dD^	80.13 ± 4.37 ^bA^
D 5000	1.33 ± 0.14 ^cC^	75.22 ± 1.57 ^cB^	1.97 ± 0.15 ^cC^	69.92 ± 3.45 ^cB^
C 500	1.98 ± 0.12 ^bB^	62.96 ± 3.42 ^dC^	2.76 ± 0.17 ^bB^	57.85 ± 4.71 ^dC^
C 1000	2.33 ± 0.38 ^bB^	56.70 ± 4.60 ^eC^	3.08 ± 0.06 ^bB^	53.07 ± 1.59 ^dC^
Control	5.36 ± 0.31 ^aA^		6.57 ± 0.31 ^aA^	

Data show mean values ± SD. Different small and capital letters indicate significant differences at 5% (*p* < 0.05) and 1% (*p* < 0.01) levels, respectively.

**Table 2:** The influences of difenoconazole and chitosan on the ash, extractum, polysaccharide, and total saponins contents in *P. heterophylla* roots.

Treatments	Ash (%)	Extractum (%)	Polysaccharide (%)	Total Saponins (%)
D 5000 + C 500	3.46 ± 0.05 ^a^	3.46 ± 0.05 ^a^	10.17 ± 0.33 ^a^	4.75 ± 0.07 ^a^
D 3000	3.37 ± 0.07 ^a^	3.37 ± 0.07 ^a^	9.38 ± 0.17 ^b^	4.44 ± 0.08 ^b^
D 5000	3.18 ± 0.08 ^b^	3.18 ± 0.08 ^b^	9.14 ± 0.13 ^bc^	4.15 ± 0.10 ^cd^
C 500	3.35 ± 0.08 ^a^	3.35 ± 0.08 ^a^	9.51 ± 0.34 ^b^	4.48 ± 0.05 ^b^
C 1000	3.21 ± 0.03 ^b^	3.21 ± 0.03 ^b^	9.13 ± 0.29 ^bc^	4.22 ± 0.09 ^c^
Control	3.15 ± 0.02 ^b^	3.15 ± 0.02 ^b^	8.68 ± 0.47 ^c^	4.06 ± 0.08 ^d^

Data show the mean value ± SD. Small letters indicate significant differences at 5% (*p* < 0.05) levels.

**Table 3:** The soil fertility of *P. heterophylla* herbal garden.

Parameters	Amount	Parameters	Amount
pH	4.93	Exchangeable calcium	19.53 cmol kg^−1^
Organic matter	35.62 g kg^−1^	Exchangeable magnesium	287.54 mg kg^−1^
Alkali hydrolyzed nitrogen	113.68 mg kg^−1^	Available zinc	2.02 mg kg^−1^
Available phosphorus	56.37 mg kg^−1^	Available iron	8.43 mg kg^−1^
Available potassium	129.56 mg kg^−1^	Available manganese	18.27 mg kg^−1^

## Data Availability

The datasets used or analyzed during the current study are available from the corresponding author upon reasonable request.
